# Evolving treatment strategies for EGFRex20ins-mutated NSCLC: a comprehensive review of Amivantamab’s role and future directions

**DOI:** 10.3332/ecancer.2025.2037

**Published:** 2025-11-17

**Authors:** Rafael Alvim Pereira, Milena Tumelero, Wallace Klein Schwengber, Gabriel Lenz

**Affiliations:** 1 Department of Medicine, Hospital Santa Casa, São José dos Campos, SP 12210-110, Brazil; 2 Department of Medicine, UNOESC University, Joaçaba, SC 89600-000, Brazil; 3 Mayo Clinic Cancer Center, Phoenix, AZ 85050, USA; 4 Internal Medicine, AdventHealth, Orlando, FL 32804, USA

**Keywords:** non-small cell lung cancer, EGFR exon 20 insertion, EGFRex20ins, amivantamab, bispecific antibody, MET, mobocertinib, targeted therapy, central nervous system metastases, drug resistance

## Abstract

Epidermal growth factor receptor exon 20 insertion mutations (EGFRex20ins) are a unique molecular subtype of non-small cell lung cancer (NSCLC) linked to resistance to EGFR tyrosine kinase inhibitors of the first and second generations. Until recently, these patients had few and frequently ineffective treatment options. Amivantamab, a bispecific antibody targeting both EGFR and mesenchymal–epithelial transition factor, has become a novel therapeutic strategy for this population. This review explores the mechanism of action of Amivantamab and its clinical efficacy and safety as demonstrated in clinical trials. Additionally, the clinical development of the subcutaneous formulation of amivantamab, real-world evidence and its regulatory status were evaluated. Lastly, we contextualise Amivantamab in the current treatment landscape by contrasting it with mobocertinib and highlighting current studies that aim to improve central nervous system activity and overcome resistance mechanisms. This review highlights the therapeutic benefit of Amivantamab in EGFRex20ins-mutated NSCLC and offers guidance for future research in this quickly developing area.

## Introduction

Lung cancer is still one of the most prevalent cancers in the world and the US, and socioeconomic and geographic factors affect how often it occurs [1]. It remains a significant public health concern even though rates in the United States have been steadily falling, particularly among men since the early 1990s and among women more recently [1–3]. 85% of primary lung tumours are non-small cell lung cancer (NSCLC), and many patients receive a diagnosis at an advanced stage. Palliative systemic therapy is frequently the cornerstone of treatment for these patients [4]. Nevertheless, since 2004 for epidermal growth factor receptor (EGFR)-mutated disease and 2011 for anaplastic lymphoma kinase-rearranged disease, patients with these oncogenic drivers can receive targeted therapies that are less harmful and more effective than chemotherapy, thanks to advancements in molecular diagnostic techniques [4–6].

More than 600 distinct EGFR mutation variants have been found due to the heterogeneity of NSCLC. Up to 40% of NSCLC cases have driver mutations in EGFR exons 18 to 21. Of these, approximately 85% of EGFR-positive cases have point mutations in exons 18, 19 and 21, which are known to react favourably to EGFR-targeted tyrosine kinase inhibitors (TKIs). Exon 20 insertions (exon20ins) are the second most frequent EGFR alteration, occurring in about 12% of cases [7–9].

Because they usually do not respond well to standard chemotherapy and immunotherapy and are resistant to conventional EGFR TKIs, EGFR exon20ins in NSCLC present unique clinical challenges [10, 11]. Amivantamab has shown encouraging potential as treatment for these mutations by bindings to the extracellular domains of EGFR and mesenchymal–epithelial transition factor (MET), enabling it to overcome resistance mechanisms associated with conventional TKIs, which target the intracellular kinase domain [12, 13].

## Molecular and therapeutic rationale for Amivantamab

Insertion mutations in exon 20 of the EaGFR gene are resistant to traditional TKIs due to an altered conformation at the kinase active site. Furthermore, the EGFR and MET genes are highly expressed in NSCLC but are rarely co-expressed in healthy cells, promoting oncogenic signaling and tumour microenvironment remodeling [14–17]. Alterations in these pathways are among the most common mechanisms of resistance to EGFR TKIs.

Amivantamab was first approved and used in the US on 21 May 2021, for the treatment of adult patients with locally advanced or metastatic NSCLC harboring EGFR exon 20 insertion mutations whose disease had progressed on or after platinum-based chemotherapy [18]. Amivantamab is a fully human monoclonal antibody of the IgG1 Fc-active type, with bispecific specificity for EGFR and MET, developed using Genmab’s DuoBody technology. The drug has two distinct arms: one binds to the extracellular domain of EGFR, blocking interaction with the epidermal growth factor (EGF), while the other prevents binding to the MET receptor [7, 9]. This dual inhibition simultaneously blocks EGFR and MET signaling and is effective against resistance mechanisms to EGFR-targeted therapy mediated through the MET pathway. This unique structural design enables Amivantamab to eliminate antigen-expressing tumour cells through antibody-dependent cellular cytotoxicity, as well as antibody-dependent cellular phagocytosis and cytokine release. This activity leads to endocytosis of the receptor-antibody complex and its removal via lysosomal trafficking [19]. By targeting both receptors, Amivantamab provides a novel therapeutic approach for overcoming resistant mechanisms in these cancer types [20].

**Figure 1.**
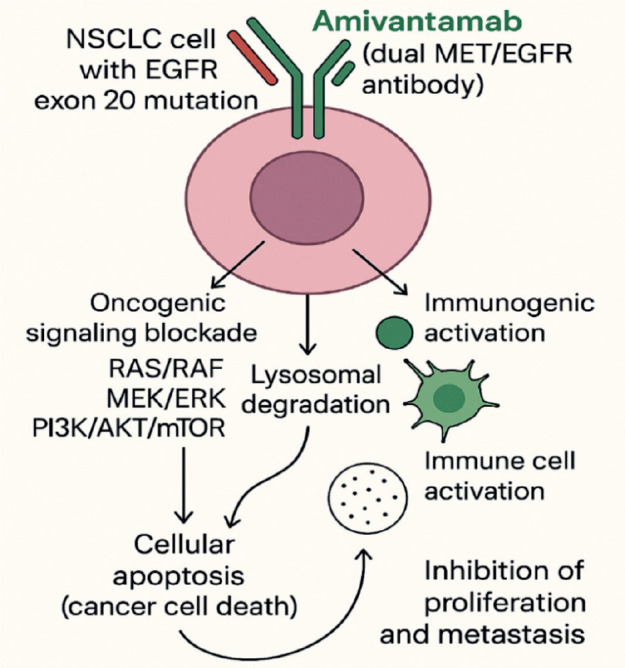
Amivantamab’s mechanism of action. Original figure.

In preclinical studies, it was demonstrated that Amivantamab binds to the extracellular domains of the EGFR and MET receptors with binding affinities of 1.43 and 0.04 nM, respectively [20]. EGFR exon20ins mutations are a heterogeneous group of alterations that occur within the C-terminal portion of the tyrosine kinase domain, most commonly between codons 762 and 774. These insertions can be broadly classified into near-loop (within or adjacent to the C-helix, e.g., A763_Y764insFQEA) and post-alpha-helix (in the loop following the C-helix, e.g., D770_N771insNPG or H773_V774insNPH) subtypes [21]. In Ba/F3 cell lines containing mutations in EGFR exon20ins, Amivantamab reduced the expression of EGFR and MET and inhibited cell viability in a dose-dependent manner in five exon20ins variants (V769_D770insASV, D770delinsGY, H773_V774insH, Y764_V765insHH and D770_N771insSVD) [12]. The mechanism of action involved the inhibition of proliferative signaling pathways (pERK, pAkt and p-S6) and the induction of apoptosis via regulation of pro-apoptotic proteins such as Bcl-2–like protein 11 and cleaved caspase-3 [19]. Amivantamab was selected from a panel of bispecific anti-EGFR and anti-MET molecules with low fucose levels in the Fc region, which increases its affinity for immune effector cells, thereby enhancing immune-mediated antitumour activity. This combination of properties gives Amivantamab the potential to increase both the depth and duration of the therapeutic response in patients with EGFR mutations, particularly those associated with resistance to TKIs [22]. In addition to its favourable mechanism of action, pharmacokinetic data from vivo trials demonstrated that Amivantamab exposure increased proportionally at doses ranging from 350 to 1,750 mg. The drug exhibited a half-life of 11.3 (± 4.53) days and a median volume of distribution of 5.13 L. Furthermore, no clinically significant differences in exposure were observed across variables such as age, sex, race, creatinine clearance or hepatic impairment [19, 20]. Figure 1 below summarises the mechanism of action of Amivantamab.

## Clinical evidence: efficacy and safety in EGFR exon20ins NSCLC

### CHRYSALIS

In the first-in-human, open-label, phase 1 CHRYSALIS trial (NCT02609776), patients with advanced NSCLC who had EGFR exon20ins mutations—a subgroup known to respond poorly to conventional EGFR inhibitors—were assessed using Amivantamab. This non-randomised, multicenter study enrolled 114 patients in the EGFR exon20ins cohort who had progressed on platinum-based chemotherapy. Amivantamab was given intravenously to patients at a recommended phase 2 dose of 1,050 mg (or 1,400 mg for patients weighing more than 80 kg) once a week for the first 4 weeks and then every 2 weeks after that. The trial found that the objective response rate (ORR) among 81 patients who had progressed following platinum-based chemotherapy was 40% (95% CI, 29–51), with a median duration of response of 11.1 months (95% CI, 6.9–not reached) and a median progression-free survival (PFS) of 8.3 months (95% CI, 6.5–10.9). Responses were seen across various exon20ins variants, and the disease control rate (DCR) was 74%. In terms of safety, the most frequent adverse events associated with treatment were rash (86%), infusion-related reactions (IRRs) (66%), paronychia (45%) and stomatitis (21%). Most of these events were grades 1 or 2 and easily controlled. Notably, premedication and split-dosing techniques successfully managed IRRs, which mostly happened with the first dose [12]. These results demonstrate that Amivantamab is a promising treatment option with long-lasting clinical benefits for a patient population with limited options for targeted therapy, and they led to its expedited Food and Drug Administration (FDA) approval in 2021 [18].

### PAPILLON trial

A phase III randomised study called PAPILLON trial (NCT04538664) compared 308 patients 1:1 to chemotherapy alone (155) versus Amivantamab plus carboplatin and pemetrexed chemotherapy (153) in patients with advanced NSCLC who had EGFR exon20ins mutations. The addition of Amivantamab resulted in a significant improvement in PFS: 11.4 months compared to 6.7 months in the group receiving chemotherapy alone (hazard ratio 0.40; 95% CI, 0.30–0.53; *p* < 0.001). The study also found that the combination arm had a significantly higher ORR than chemotherapy alone [23].

PAPILLON offered strong evidence for the drug’s use in the first-line setting, helping to establish a new standard of care for patients with EGFR exon20ins-mutant NSCLC, while CHRYSALIS demonstrated the drug’s effectiveness as a second-line option [12, 23, 24]. Table 1 below summarises the Key Clinical Trials in EGFR exon20ins-Mutant NSCLC.

### Safety profile and IRRs

The safety profile of Amivantamab has been well documented in the CHRYSALIS and PAPILLON trials, particularly concerning IRRs, which occurred in about 66% of patients in the CHRYSALIS trial—most commonly during the first infusion. Chills, shortness of breath, flushing, nausea, chest pain and vomiting were typical symptoms of these reactions, which were typically mild to moderate in severity (grades 1 or 2). Antihistamines and antipyretics were usually prescribed to patients in advance to treat these occurrences and infusion rates were changed as needed [12, 18, 25].

The PAPILLON trial assessed Amivantamab in combination with chemotherapy and reported comparable IRR. These IRRs were primarily associated with the initial dose and decreased with subsequent infusions [10].

IRRs are among the most common side effects of Amivantamab, but with the right premedication and infusion procedures, they are usually controllable. Although these side effects rarely disrupt continued therapy, they emphasise how crucial close observation is during the initial dosage to guarantee patient safety and treatment continuity [18, 25]. Table 2 below summarises the adverse effects across trials.

## The subcutaneous (SC) shift: clinical and operational impact

### PALOMA-3 trial

A SC form of Amivantamab was created to preserve comparable pharmacokinetics and provide a more practical option for intravenous (IV) administration. The international, randomised, open-label Phase III PALOMA-3 trial (NCT05388669) aimed to determine whether SC versus IV Amivantamab administration, both in combination with lazertinib, was non-inferior in patients with advanced NSCLC that displayed EGFR

exon 19 deletions or L858R mutations and had advanced following previous treatment with osimertinib and platinum-based chemotherapy. A total of 418 patients were randomly assigned in a 1:1 ratio to receive Amivantamab intravenously (*n* = 212) or subcutaneously (*n* = 206), each in addition to daily oral lazertinib at a dose of 240 mg. The study achieved its primary goal by proving the SC formulation’s pharmacokinetic non-inferiority. The median PFS was 6.1 months for SC and 4.3 months for IV, while the ORR was similar for both arms (30% for SC and 33% for IV). Crucially, SC administration led to a five-fold decrease in venous thromboembolism incidence (9% versus 14%) and IRR (13% versus 66%). The SC group benefited from significantly shorter administration times (median 4.8 minutes versus 5 hours), but the treatment duration was comparable (median 7.2 versus 7.0 months). According to these results, SC amivantamab is safer, more practical and more efficient than IV delivery [26].

Amivantamab’s SC formulation preserves pharmacokinetic equivalency, improves patient convenience and satisfaction and offers significant advantages in operational efficiency and possible cost savings [27].

### FDA rejection (2024)

Despite all its potential benefits, the SC form of Amivantamab (Rybrevant), which was being evaluated for patients with EGFR-mutated NSCLC, was rejected by the FDA in October 2024. Problems discovered during a routine manufacturing facility inspection served as the basis for the decision. Crucially, no additional clinical trials were needed, and the FDA expressed no concerns regarding the drug’s safety, efficacy or formulation [28].

### EMA approval (2025)

The SC formulation of Amivantamab (Rybrevant) and lazertinib (Lazcluze) was approved by the European Commission Agency in April 2025 for use as a first-line treatment for adults with advanced NSCLC that has either exon 21 L858R mutations or EGFR exon 19 deletions. For patients with advanced NSCLC who have EGFR exon20ins mutations and have advanced following platinum-based chemotherapy, it was also authorised as a monotherapy [29].

### Equity and access: challenges in low-resource systems

Some things are important to be considered depending on the healthcare setting. The SC route offers different advantages: first, it diminishes costs and time of administration, which could impact patient satisfaction. This can lead to a reduced financial burden on healthcare systems. These factors are very important to consider because some places, such as busy clinics or rural settings, have high demand and time is crucial. Although SC route offers great advantages, IV administration may be needed when the patient must be closely monitored, such as when early treatment of other IV drugs is being administrated [30].

Access and equity remain significant obstacles, particularly in low-resource environments where advanced therapies may be difficult to deliver due to supply chain problems, high treatment costs and inadequate healthcare infrastructure. Amivantamab and other SC formulations may lessen the logistical load, but safe administration requires trained personnel and appropriate storage. Since the high cost of targeted treatments also raises important questions about equity and access globally, stronger international health regulations and support networks are needed to make precision oncology more accessible to everyone, regardless of where they live [30].

## Integration into clinical practice: NCCN guidelines

In the NCCN Guidelines for NSCLC, Amivantamab (Rybrevant) has taken center stage. Several Category 1 and 2A recommendations are specific to EGFR mutations and treatment contexts. Amivantamab combined with carboplatin and pemetrexed is now a Category 1 preferred first-line treatment for patients with newly diagnosed advanced or metastatic nonsquamous NSCLC that has EGFR exon20ins mutations.

According to the MARIPOSA-2 trial (NCT04988295), Amivantamab plus chemotherapy, with or without lazertinib, is also a Category 1 subsequent therapy for patients with EGFR exon 19 deletions or exon 21 L858R mutations who do not progress on Osimertinib [31]. Moreover, amivantamab and lazertinib are now Category 1-recommended for these prevalent EGFR mutations in the first-line setting. Amivantamab can also be used on its own (monotherapy) as a Category 2A option for patients with EGFR exon20ins mutations whose cancer has progressed after platinum-based chemotherapy. These updates demonstrate the growing significance of Amivantamab as a treatment for EGFR-mutated NSCLC, providing new options for various therapy stages and mutation types [32].

## Comparative landscape and positioning of Amivantamab

### Mobocertinib

Mobocertinib (Exkivity) is an oral TKI designed to target EGFR exon20ins mutations in NSCLC, a subset known to be resistant to earlier-generation EGFR inhibitors. In 2021, the FDA gave accelerated approval for patients with metastatic or advanced NSCLC who had progressed while receiving platinum-based chemotherapy [18]. However, after the EXCLAIM-2 trial (NCT04129502) failed to demonstrate a definite advantage over conventional chemotherapy in the first-line setting, Takeda voluntarily removed the medication from the market in 2023 [33–35]. Although there was a lot of interest in mobocertinib at first, especially given how challenging EGFR exon20ins mutations are to treat—its modest effectiveness and side effects like diarrhea and QTc prolongation ultimately kept it from becoming a widely used option.

### Sunvozertinib

An oral, third-generation, irreversible EGFR TKI called sunvozertinib is presently being developed to treat NSCLC with mutations in the EGFR exon20ins. It has demonstrated promising outcomes in patients who had previously received platinum-based chemotherapy. According to data from the pivotal WU-KONG6 trial (NCT05712902), 97 patients had a confirmed ORR of 59.8%, with 48.4% having baseline brain metastases [36]. Early-phase studies indicate a median PFS of approximately 8–9 months, and DCRs have been reported to be between 80% and 90% [37, 38]. In addition, sunvozertinib has demonstrated positive brain activity. Compared to amivantamab, it is a more convenient option because it is taken orally once daily. With common side effects like rash, diarrhea and paronychia, the safety profile is generally good [39]. Although both medications are equally effective (amivantamab has an ORR of about 40%), amivantamab must be administered intravenously and has limited central nervous system (CNS) penetration and infusion-related side effects. Sunvozertinib is a viable and practical therapeutic choice that is still being assessed in ongoing clinical trials [32].

### Poziotinib

Poziotinib is an oral irreversible TKI that targets EGFR and human epidermal growth factor receptor 2 (HER2) exon20ins mutations in NSCLC. In the ZENITH20 trial (NCT03066206), the ORR ranged from 27% to 32%, with DCRs of nearly 70% in previously treated patients. Early-phase studies also revealed positive indications of activity [40, 41]. However, the drug’s limited effectiveness and frequent side effects ultimately led to its development being shut down. Many patients had unpleasant side effects like rash, diarrhea and mouth sores, which frequently required changing the dosage or discontinuing treatment completely [41]. Despite the benefit of being taken orally, poziotinib was withdrawn from FDA review and further development because it could not provide a robust enough risk-benefit profile.

In conclusion, amivantamab presently provides a clinically proven, validated and beneficial treatment option, particularly when used in conjunction with chemotherapy. However, the changing landscape—especially with drugs like sunvozertinib—may reshape treatment norms by providing improved CNS activity, oral administration convenience and equal or better efficacy. Table 3 below the efficacy, safety and development status of targeted therapies for ECGFR exon20ins-Mutant NSCLC.

### Real-world outcome data (2024–2025 studies)

The efficacy of Amivantamab in treating NSCLC with EGFR exon20ins mutations has been further supported by real-world data from 2024 to 2025. The 2025 American Society of Clinical Oncology Living Guideline emphasised the potential of combination therapies, pointing out that in patients with high-risk characteristics, like TP53 co-mutations, Amivantamab plus lazertinib enhanced PFS in comparison to osimertinib alone [43].

In one study, Wang *et al* [44] found that patients from various cancer centers had a DCR of 64.3% and a clinical response rate of 35.7%. Even when Amivantamab was used in conjunction with radiation therapy or osimertinib, the safety profile stayed the same and no new toxicities were observed [44].

The Lung Cancer Genomic Screening Project for Individualized Medicine in Japan-Asia study also provided additional information by demonstrating that the location of exon20ins may impact treatment outcomes. When Amivantamab was administered to patients instead of docetaxel, immune checkpoint inhibitors or classical TKIs, the patient’s overall survival (OS) and PFS improved [45].

These results demonstrate that amivantamab is a potent treatment option for patients with EGFR exon20ins mutations because it is practical and well-tolerated, particularly when combined with other targeted therapies or customised to the patient’s risk factors.

## Future directions and unanswered questions

### Resistance mechanisms to amivantamab

Although the exact mechanism of amivantamab resistance in NSCLC with EGFR exon20ins mutations is still unknown, information from comparable EGFR-targeted therapies provides some insight. By attaching to the extracellular domains of EGFR and MET, the bispecific antibody amivantamab can help get around some of the resistance that TKIs cause [9, 12]. However, there are a few ways that resistance can arise. One potential mechanism involves secondary mutations in EGFR that alter the receptor’s shape and decrease the drug’s binding ability.

Another possible pathway is MET amplification, which permits cancer cells to depend more on MET signaling even when EGFR is inhibited [46]. To avoid EGFR and MET, tumours may activate other survival pathways, such as those involving HER2 or HER3 [47]. Finally, histologic transformation—where the tumour changes type, for instance, becoming small cell lung cancer—is a known resistance mechanism with other EGFR therapies and may also occur here, even though it has not been documented with amivantamab specifically yet. As we seek methods to get past resistance and enhance amivantamab results, it is critical to comprehend these potentialities.

The MET pathway acts as a compensatory mechanism to EGFR signaling, with MET amplification being a well-established mechanism of resistance to EGF receptor TKIs. However, it remains unclear whether this resistance mechanism also applies to TKIs specifically targeting EGFR exon20ins. Amivantamab, a bispecific EGFR/MET antibody, stands out for its dual mechanism of action and has shown efficacy in patients with EGFR mutations who have progressed after osimertinib therapy [48, 49].

The exploration of anti-MET antibody therapies represents a promising approach in the treatment of NSCLC, particularly in the context of resistance to EGFR inhibitors. Dysregulation of the MET pathway, through overexpression, exon 14 mutation or gene amplification, plays a critical role in oncogenesis and therapeutic resistance. While the METex14 mutation is the most validated biomarker, MET amplification, particularly assessed by the MET/CEP7 ratio using FISH, demonstrates superior predictive potential [50].

Another point is amivantamab’s possible activity outside of this subgroup of exon 20. G719X, S768I, L861Q and specific exon 18 insertions are examples of uncommon EGFR mutations that are typically less sensitive to early-generation TKIs but may still be partially sensitive to third-generation agents. Preclinical research has demonstrated that amivantamab’s bispecific EGFR/MET binding is effective against some rare variants, possibly circumventing resistance resulting from changed kinase conformations [51].

Furthermore, complex resistance patterns and distinct biological behaviour can be conferred by compound EGFR mutations, which are defined as two or more EGFR alterations in the same tumour. Early clinical experience and retrospective reports indicate that amivantamab—alone or paired with lazertinib—may help some patients with compound EGFR mutations, particularly when one of the alterations is an exon20ins. However, because these genotypes are highly diverse and treatment responses can vary, prospective studies are needed to better define which patients are most likely to benefit [44].

Importantly, strategies to overcome or delay resistance are under investigation. For instance, in patients with EGFR exon20ins–mutated NSCLC, amivantamab added to carboplatin–pemetrexed chemotherapy in the first-line setting significantly extended PFS when compared to chemotherapy alone (PAPILLON trial) [23]. Furthermore, real-time early detection of new resistance mechanisms is made possible by liquid biopsy, which includes circulating tumour DNA analysis [52]. This allows for prompt therapeutic adaptation and may extend clinical benefit.

The future of NSCLC treatment will depend on the precise integration of biomarkers, optimisation of the safety of combination therapies and further understanding of MET pathway biology. Ongoing clinical trials and translational studies will be crucial in consolidating these therapies into clinical practice [49].

## Conclusion

The treatment of NSCLC with EGFR exon20ins has been entirely transformed by the discovery of amivantamab, a more effective alternative to traditional chemotherapy. As research into its use in different combinations and environments continues, its role in treating this troublesome form of lung cancer is becoming increasingly apparent. More recently, the development of an SC formulation has increased the appeal of treatment by making it quicker and simpler to administer. Compared to IV infusion, SC delivery improves the overall quality of treatment, takes only a few minutes and minimises side effects associated with infusion. This change relieves the burden on infusion facilities and employees while promoting more adaptable care models, such as outpatient and possibly even home-based options. Because SC delivery eliminates the need for specialised infrastructure, it may also increase access in rural or resource-constrained areas. This strategy aligns nicely with the increasing emphasis on patient-centered, value-based cancer care. As SC amivantamab is used more frequently, it may improve the treatment of EGFR-mutated NSCLC and influence the way targeted therapies are administered in oncology in the future. Nonetheless, resistance mechanisms remain a vital frontier, with ongoing studies exploring optimal sequencing, novel combinations and activity across broader

EGFR mutation subtypes. Amivantamab’s therapeutic footprint is anticipated to grow as empirical data mount and biomarker-driven methods improve patient selection, impacting not only the treatment of EGFR-mutated NSCLC but also the delivery models of targeted therapies in oncology going forward.

## Disclosure

Rafael Alvim Pereira has no disclosures. Milena Tumelero has no disclosures. Wallace Klein Schwengber has no disclosures. Gabriel Lenz has no disclosures.

## Conflicts of interest

The authors Rafael Alvim Pereira, Milena Tumelero, Wallace Klein Schwengber and Gabriel Lenz declare that they have no conflicts of interest related to this work.

## Funding

No funding was received for the publication of this article.

## Figures and Tables

**Table 1.** Comparison of key clinical trials in EGFR exon20ins–mutant NSCLC [12, 23].

Trial name	*N*	CNS	ORR (%)	Median PFS (months)	Median OS (months)
**CHRYSALIS** (NCT02609776)	114 (exon 20 cohort)	Limited data	40% (in EGFR exon20ins)	8.3	22.8 months
**PAPILLON** (NCT04538664)	308	Included, stratified	47% (chemo) versus 73% (Ami + chemo)	6.7 (chemo) versus 11.4 (Ami + chemo)	Not reported yet (interim)

Ami = amivantamab; Chemo = chemotherapy; CNS = central nervous system; EGFR = epidermal growth factor receptor; NR = not reported; NSCLC = non-small-cell lung cancer; ORR = objective response rate; OS = overall survival; PFS = progression-free survival. Original table

**Table 2.** Adverse effects across trials [12, 23].

Trial name	Grade ≥3 AEs (%)	Notable specific AEs
**CHRYSALIS** (NCT02609776)	35% (Amivantamab monotherapy)	Rash (86% all-grade, 3% grade ≥3), IRRs (66%, mostly grade 1–2), paronychia (45%)
**PAPILLON** (NCT04538664)	Ami + Chemo: 75% versus Chemo: 54%	Neutropenia (59% all grade, 33% grade ≥3), rash (54% all-grade, 11 grade ≥3), paronychia (56%)

AEs = adverse effects; Ami = amivantamab; Chemo = chemotherapy. Original table

**Table 3.** Efficacy, safety and development status of targeted therapies for EGFR exon20ins–mutant NSCLC [12, 36, 41, 42].

Drug (Trial identifier)	Type	ORR (%)	Median PFS (months)	CNS Activity	Route	Key AEs (≥ Grade 3)	Development Status
**Amivantamab** (NCT02609776)	Bispecific EGFR-MET monoclonal antibody	40	8.3	Limited	IV infusion	Rash (4%), IRR (3%), Paronychia (1%)	FDA-approved; ongoing phase III trials
**Mobocertinib** (NCT02716116)	Oral EGFR TKI	28	7.3	Limited	Oral	Diarrhea (16%), QTc prolongation (3%)	Withdrawn from market (2023)
**Sunvozertinib** (NCT05712902)	Oral EGFR TKI (3rd-gen, irreversible)	61	7.6	Promising	Oral	Blood creatine Phosphokinase increased (17%), Diarrhea (8%), Anemia(6%)	In development; ongoing trials
**Poziotinib** (NCT03318939)	Oral EGFR/HER2 TKI	27.8	5.5	Limited	Oral	Rash (48%), Diarrhea (25%), Stomatitis (24%)	Development discontinued

EGFR = Epidermal Growth Factor Receptor; NSCLC = Non–Small Cell Lung Cancer; ORR = Objective Response Rate; PFS = Progression-Free Survival; CNS = Central Nervous System; IV = Intravenous; IRR = Infusion-Related Reaction; TKI = Tyrosine Kinase Inhibitor

Adverse events (AEs) reported are grade ≥3 where available. Data are derived from single-arm clinical trials unless otherwise specified. Trial identifiers (NCT numbers) correspond to ClinicalTrials.gov registry entries.

Note: Mobocertinib data are based on the original EXCLAIM trial (NCT02716116) in previously treated patients; data from EXCLAIM-2 are not included in this table. Original table
